# Health-Related Quality of Life in Children and Adolescents with Hereditary Bleeding Disorders and in Children and Adolescents with Stroke: Cross-Sectional Comparison to Siblings and Peers

**DOI:** 10.1155/2016/1579428

**Published:** 2016-05-15

**Authors:** Bruno Neuner, Sylvia von Mackensen, Susanne Holzhauer, Stephanie Funk, Robert Klamroth, Karin Kurnik, Anne Krümpel, Susan Halimeh, Sarah Reinke, Michael Frühwald, Ulrike Nowak-Göttl

**Affiliations:** ^1^Department of Anesthesiology and Intensive Care Medicine, Charité-Universitaetsmedizin Berlin, Campus Charité Mitte and Campus Virchow-Klinikum, 10117 Berlin, Germany; ^2^Institute for Medical Psychology, University Medical Center Hamburg-Eppendorf, 20246 Hamburg, Germany; ^3^Department of Pediatric Hematology/Oncology, Charité-Universitaetsmedizin Berlin, Campus Virchow-Klinikum, 13353 Berlin, Germany; ^4^Working Group Cardiovascular Magnetic Resonance, Experimental und Clinical Research Center (ECRC), Charité-Universitaetsmedizin Berlin, Campus Buch, 13125 Berlin, Germany; ^5^Department of Internal Medicine, Angiology and Clotting Disorders, Haemophilia Treatment Centre, Vivantes Clinic Friedrichshain, 10249 Berlin, Germany; ^6^Pediatric Thrombosis and Hemostasis Unit, Dr. von Hauner Children's Hospital, Medical Center of the University of Munich, 80337 Munich, Germany; ^7^Department of Pediatric Hematology/Oncology, University of Münster, 48149 Münster, Germany; ^8^Coagulation Centre Rhine-Ruhr, 47051 Duisburg, Germany; ^9^Department of Clinical Chemistry, University Hospital Schleswig Holstein, 24105 Kiel, Germany; ^10^Children's Hospital, Department of Pediatric Oncology and Hematology, 86156 Augsburg, Germany

## Abstract

*Objectives*. To investigate self-reported health-related quality of life (HrQoL) in children and adolescents with chronic medical conditions compared with siblings/peers.* Methods*. Group 1 (6 treatment centers) consisted of 74 children/adolescents aged 8–16 years with hereditary bleeding disorders (HBD), 12 siblings, and 34 peers. Group 2 (one treatment center) consisted of 70 children/adolescents with stroke/transient ischemic attack, 14 siblings, and 72 peers. HrQoL was assessed with the “revised KINDer Lebensqualitätsfragebogen” (KINDL-R) questionnaire. Multivariate analyses within groups were done by one-way ANOVA and post hoc pairwise single comparisons by Student's *t*-tests. Adjusted pairwise comparisons were done by hierarchical linear regressions with individuals nested within treatment centers (group 1) and by linear regressions (group 2), respectively.* Results*. No differences were found in multivariate analyses of self-reported HrQoL in group 1, while in group 2 differences occurred in overall wellbeing and all subdimensions. These differences were due to differences between patients and peers. After adjusting for age, gender, number of siblings, and treatment center these differences persisted regarding self-worth (*p* = .0040) and friend-related wellbeing (*p* < .001).* Conclusions*. In children with HBD, HrQoL was comparable to siblings and peers. In children with stroke/TIA HrQoL was comparable to siblings while peers, independently of relevant confounder, showed better self-worth and friend-related wellbeing.

## 1. Introduction

Hereditary bleeding disorders (HBD) encompass a heterogeneous group of diseases with the most frequent clinical entities being von Willebrand disease (vWD) and hemophilia A (HA). Their incidence is overall low but the individual burden of disease remains high despite encouraging advances in secondary prevention including prophylactic factor replacement and enhanced treatment options such as optimized joint disease prevention and improved inhibitor management [1–4]. When patients with HA receive optimal medical care and when they are not affected by blood-borne viruses (such as HIV or HCV) they may face a life expectancy similar to that of the general population [5, 6]. It remains unclear whether quantitative gains in terms of life expectancy are accompanied by qualitative gains in self-reported health-related quality of life (HrQoL) whether these gains are apparent already in young persons with HA. Recent studies found in children and adolescents with HA and other HBD HrQoL comparable to or even better than population norms. Poon et al. [7] found in their study in Californian children with HA (mean age 9.7 years) HrQoL (assessed with the generic PedsQL) similar to or better than population norms. Likewise Bullinger et al. [8] found generic HrQoL in more than 300 children with hemophilia in 6 European countries higher compared to children with other chronic medical conditions. Gringeri et al. [9] assessed HrQoL in 20 treatment centers in 6 European countries using the hemophilia-specific Haemo-QoL. The 4–16-year-old patients with hemophilia A and hemophilia B showed solely impairments in family-related wellbeing in 4–7-year-old patients, whereas 8–16-year-old patients showed impairments in friend-related wellbeing compared to younger patients [9]. The total score of the Haemo-QoL questionnaire showed no overall differences between age groups but differences between treatment groups (on-demand treatment versus prophylactic treatment). Alpkiliç Baskirt et al. [10] compared a group of 71 patients with HA, 14–34-year-old, matched for age and gender to healthy controls and found in five out of eight domains of the SF-36 significantly higher scores in healthy controls compared to the patient group. A similar approach was chosen by Salem and Eshghi [11] from Tehran who investigated 46 patients with HBD aged 2–15 years. These patients were likewise compared to age and gender matched controls. Here the authors found no differences between patients and controls regarding oral HrQoL (using the Child Perception Questionnaire, CPQ, items 1–9) as well as regarding functional limitations, emotional and social wellbeing (CPQ, items 10–24).

However, findings were compared between subgroups of patients or between hemophilic patients and patients with other chronic medical conditions or with population norm data. Similarly a recent narrative review of studies evaluating self-reported quality of life in children with various medical conditions found most investigators comparing patients' self-reports with general population or age-matched control/reference children [13].

To the best of our knowledge few studies have investigated HrQoL in children with HBD in comparison with their siblings or with healthy individuals deriving from their social environment. Therefore aim of the current investigation was to compare self-reported measures of HrQoL in a group of children and adolescents with a chronic medical condition but no expected functional restrictions, HBD, to their siblings and peers. Data from an own investigation in children with stroke/transient ischemic attack (TIA) allowed repeating these comparisons between patients, siblings, and peers in a disease group with neonatal or childhood stroke at a median age of 6.3 years [14]. The majority of these children exhibited at least one moderate neurological deficit with functional restrictions [14].

## 2. Material and Methods

### 2.1. Inclusion Criteria

Inclusion criteria were as follows: pediatric patients with HBD, aged 8 to 16 years, admitted to 6 specialized treatment centers in Germany. Patients were recruited during their routine outpatient's visits in clinics in major cities in Germany (2 centers in Berlin [*n* = 36], Duisburg [*n* = 10], Leipzig [*n* = 3], Münster [*n* = 15], and Munich [*n* = 11]).

### 2.2. Exclusion Criteria

Exclusion criteria were as follows: patients aged less than 8 years at the time of completion of the questionnaire, non-German speaking families, children who were unable to understand and answer the questions due to severe disabilities, and families without written informed consent.

### 2.3. Study Population

Study participants were enrolled between August 2008 and August 2011 (patients with HBD) and in January 2010 (patients with TIA/stroke). Out of 354 initial study participants 37 were older than 17 years (10.5%). Of the remaining 299 study participants, 23 did not fill out the “revised KINDer Lebensqualitätsfragebogen” (KINDL-R, [15]). Thus the final dataset consists of 144 patients. Of these 74 were patients with HBD (51.4%) and 70 were patients with stroke or TIA (48.6%). The 74 patients with HBD were patients with hemophilia A and hemophilia B (*n* = 36), patients with von Willebrand disease type 2 and type 3 (*n* = 22), patients with hereditary fibrinogen deficiency (*n* = 5), and patients with factor V, VII, and XI deficiency (*n* = 9). Of these, 12 healthy siblings were willing to participate in this cross-sectional investigation. Additionally, data were collected from 34 healthy unrelated peers recruited from the same catchment areas as the patients [kindergarten/school mates and friends]. Data from patients with stroke or TIA, their siblings (*n* = 14), and peers (*n* = 72) were derived from a previous investigation at the University of Münster [14].

Patients completed the questionnaires during a routine ambulatory follow-up visit (i) by themselves or (ii) if unable to read and write with the help of a study nurse but not with the help of their parents or other accompanying persons.

### 2.4. HrQoL Outcome

The “revised KINDer Lebensqualitätsfragebogen” (KINDL-R) questionnaire [15] is a generic instrument to assess HrQoL and was originally developed in German language (http://www.kindl.org/). This 24-item self-administered or proxy-report questionnaire generates a total score (overall wellbeing) as well as scores for 6 subscales (physical wellbeing, psychological wellbeing, self-worth, family-related wellbeing, friend-related wellbeing, and school-related wellbeing). Scores range from 0 to 100, with higher values indicating better Hr-QoL. The KINDL-R instrument is validated for use in healthy children and pediatric patients aged 4 to 16 years, in 3 age groups (4 to 7 years/8 to 12 years/13 to 16 years). Out of 17,000 children and adolescents participating in a representative population sample, the German Health Survey for Children and Adolescents (Studie zur Gesundheit von Kindern und Jugendlichen in Deutschland, KIGGS), more than 6,800 completed the KINDL-R questionnaire [12]. The psychometric properties for children ≥ 11 years were published in 2009 [12]. Data on the number of siblings and the current educational status were additionally collected.

### 2.5. Statistics

Metric variables are presented as mean ± standard deviation. Multivariate comparisons of HrQoL between patients, siblings, and peers were calculated using one-way ANOVA. In case of differences between groups pairwise single comparison was done by Student's *t*-test for independent subgroups. To account for multiple testing (overall 3 tests) the *p* value for this test procedure was Bonferroni-corrected to *p* < .0167. Adjusted pairwise comparisons were by hierarchical linear models with random intercepts for treatment center (Proc mixed in SAS, using the default residual maximal likelihood estimation method for the covariance parameters and a random intercept assuming an unstructured covariance matrix). Models were adjusted for age, gender, number of siblings, and school education. To account for multiple testing (overall 7 comparisons in KINDL-R total score and 6 subdimensions) the *p* value for these analyses was Bonferroni-corrected to *p* < .0071. Otherwise a *p* value < .05 was considered statistically significant. Internal consistency of the KINDL-R questionnaire was examined by Cronbach's alpha [*α*]; Cronbach's *α* values above  .70 were considered acceptable. All statistical analyses were performed using SPSS software (version 23.0, SPSS Inc., Chicago, Illinois, US) and SAS 9.2 (Cary, North Carolina, US), respectively.

## 3. Results

In patients with HBD more than 40% (22 of 52 complete datasets) were in need of prophylactic substitution and additionally 16/52 (30.89%) were in need of both prophylactic and on-demand factor substitution. Mean age of the study participants with HBD was 11.5 years while the mean age of study participants with stroke/TIA was 11.6 years (see Table 1). The majority of patients had one sibling, while 20 (27%) patients with HBD and 6 (8.6%) patients with stroke/TIA had no siblings. Less than 5% of children with HBD attended other than mainstream schools (e.g., schools for physically handicapped children). The majority of children attended secondary schools.

**Figure 1:**
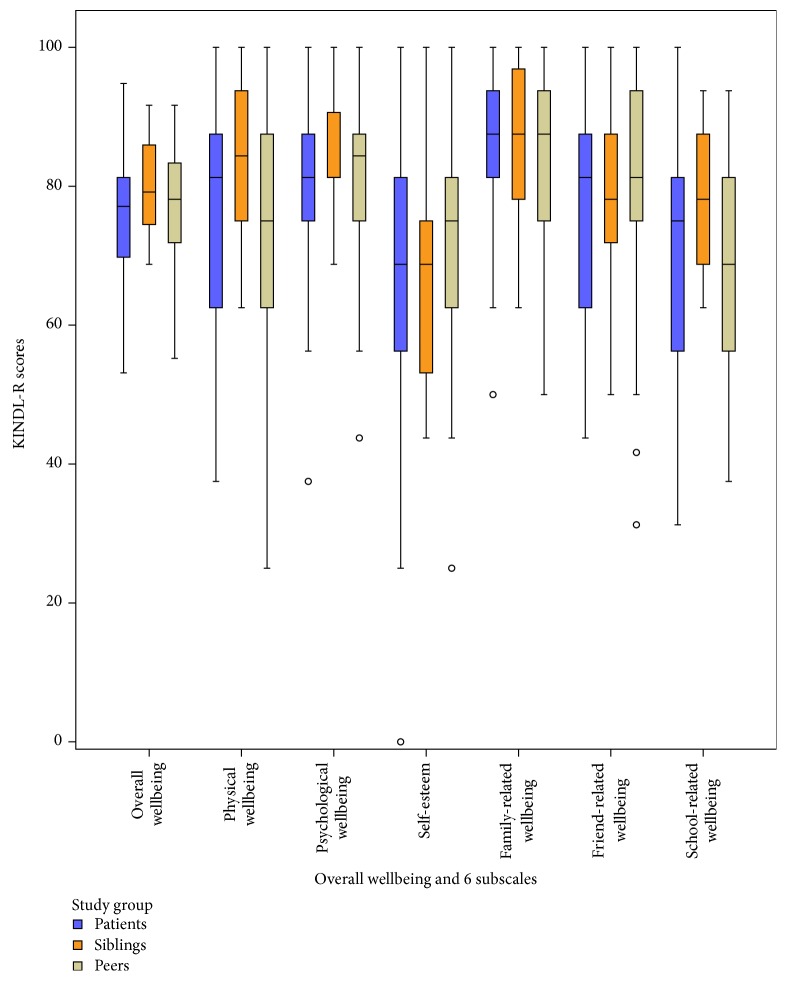
Self-reported quality of life in patients with hereditary bleeding disorders and their siblings and peers. KINDL-R: revised KINDer Lebensqualitätsfragebogen; black circles represent outliers greater than 1.5 times the interquartilrange (IQR).

**Figure 2:**
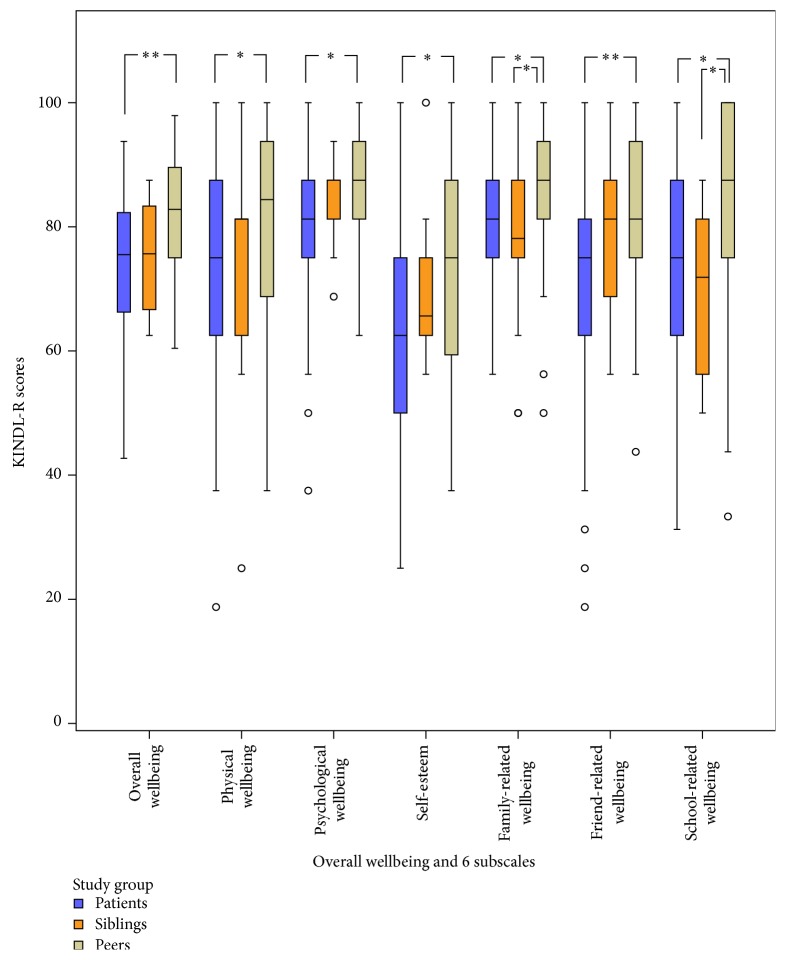
Self-reported quality of life in patients with stroke/transient ischemic attack (TIA) and their siblings and peers. KINDL-R: revised KINDer Lebensqualitätsfragebogen; ^*∗*^ *p* < .0167 (Bonferroni correction); ^*∗∗*^ *p* < .001 (results of post hoc pairwise single comparisons); black circles represent outliers greater than 1.5 times the interquartilrange (IQR).

Multivariate analyses within patients, siblings, and peers revealed no differences in self-reported overall wellbeing and all KINDL-R subdimensions in group 1 (see Figure 1; all *p* > .05). In group 2, differences occurred in multivariate analyses in self-reported overall wellbeing and all subdimensions (overall wellbeing *p* < .001, physical wellbeing *p* = .016, emotional wellbeing *p* = .007, self-worth *p* = .046, family-related wellbeing *p* = .010, friend-related wellbeing *p* < .001, and school-related wellbeing *p* = .003). When testing for pairwise differences in group 2 (see Figure 2), no differences were seen between patients and their siblings while significant differences were apparent between patient and peers in overall wellbeing and all subdimensions (all *p* < .016) and between siblings and peers regarding family-related and school-related wellbeing, respectively (both *p* < .016). In adjusted multivariate analyses, group 1 showed no differences in self-reported HrQoL between patients and siblings and between patients and peers, respectively (Table 2), while differences were apparent in group 2 between patients with stroke/TIA and their healthy peers regarding self-worth and friend-related wellbeing (Table 3).

The psychometric characteristics of the KINDL-R questionnaires, that is, the internal consistencies of self-reported KINDL-R questionnaires, are displayed in Table 4. Taking a cut-off of *α* = 0.7, overall wellbeing in both patients and healthy controls met the definition of acceptable internal consistency, as did the internal consistency in the representative German population sample, the KIGGS-study (last column in Table 4). Internal consistency of the subdimension was somewhat lower but followed pattern comparable to the population norms deriving from the KIGGS-study. Only one subdimension, friend-related wellbeing in healthy controls, showed poor results (Cronbach's *α* below 0.5). However, several other subdimensions (emotional wellbeing and school-related wellbeing) showed a reliability just above the lower threshold of a modest reliability (Cronbach's *α* between 0.5 and < 0.7). Comparison is limited by the fact that norm data were solely available for the age group of 11 years and older.

## 4. Discussion

The most relevant finding in this investigation was the overall good health-related quality of life (HrQoL)—as measured with a generic instrument, the KINDL-R questionnaire—in children with hereditary bleeding disorders. No differences in HrQoL occurred compared with their siblings. In children with stroke/TIA, differences in self-reported HrQoL were apparent in univariate analyses and related to differences between patients and their peers. These differences in self-reported HrQoL remained in multivariate models for self-worth and friend-related wellbeing. Internal consistency of the KINDL-questionnaires was good for overall wellbeing for all study groups and followed pattern of population based findings for the KINDL-R subscales.

When considering the adjusted differences between patients and siblings and patients and peers, respectively, in this investigation, observed differences in overall wellbeing were small (less than one point) indicating comparable HrQoL between groups. However, when looking for differences in HrQoL subdimensions, it appeared that differences between patients and siblings as well as between patients and peers in group 1 (hereditary bleeding disorders) were altogether less pronounced than in group 2 (stroke, TIA). Furthermore, in this group absolute differences were more pronounced in the comparison of patients and peers than in the comparison of patients and their siblings. Across all four comparisons (see Tables 2 and 3) absolute differences between groups were largest in self-worth. Choosing an arbitrary cut-off of 5 points for a clinical significant and meaningful difference between two groups, patients with stroke/TIA showed in five out of six KINDL-R domains lower HrQoL than their peers (of these two showing statistical significance either, even when applying strict constraints due to multiple testing) while there were solely two domains clinically relevant affected between patients with stroke/TIA and their siblings and only one in group 1. Thus intense medical care of patients with HBD (38 out of 52 HBD patients were in need for prophylactic and/or on-demand factor substitution) may enable young patients a quality of life similar to their sisters, brothers, and friends. The probable clinical (although in this investigation not statistical) significant impairment in self-worth in patients with HBD compared to their healthy friends may nevertheless indicate a need for psychological support and for improvement of self-esteem.

In other chronic medical conditions with no necessarily functional restrictions in daily life comparable findings were reported: no differences between HrQoL in juvenile patients with sickle cell disease and their siblings were found, but siblings scored in three out of 10 domains of the KIDSCREEN-52 questionnaire worse than Dutch population norms [16].

In children with stroke/TIA with ongoing neurological deficits in >90% [14] clinical relevant impairments were more apparent. This concerned—apart from self-worth—additional social dimensions, both in comparison to siblings and, even more pronounced, in comparison to peers. When looking at absolute values, differences were more pronounced in psychological and social domains than in physical wellbeing.

Our findings in patients with stroke/TIA reflected earlier findings from other groups of children and adolescents with chronic medical conditions leading to functional restrictions: in patients with cardiac disorders, Manlhiot et al. [17] found in 10–20-year-old patients after Fontan procedure compared with their healthy siblings impaired HrQoL in all domains of the generic Peds-QoL questionnaire. Adult survivors of Tetralogy of Fallot (224 survivors out of 1,693 repairs over a period of 44 years) showed likewise impaired general health and physical functioning (as assessed with the generic SF-36 questionnaire) compared with their siblings, especially in those with surgery at older age [18]. Likewise in patients with neurologic conditions, Mowry et al. [19] found worse HrQoL in children with multiple sclerosis (mean age 14 years) in comparison with their siblings; HrQoL was evaluated with the Pediatric Quality of Life Inventory and impairment in HrQoL was associated with neurologic deficits. Likewise impaired HrQoL in juvenile patients with other severe chronic medical conditions in comparison with their healthy siblings was found in children with cystic fibrosis [20], in children with acute lymphoblastic leukemia [21], in children with transfusion-dependent thalassemia, except for the social-relationship domain of the WHOQOL-Bref [22], and in children with hereditary retinal disorders [23].

## Figures and Tables

**Table 1:** Basic sociodemographic characteristics of all study participants with complete KINDL-R questionnaires, *n* = 276.

Variable	Patients		Healthy controls	
	Patients with HBD (*n* = 74)	Stroke or TIA (*n* = 70)	Siblings (*n* = 26)	Peers (*n* = 106)
Female gender, *n* (%)	11 (14.9%)	44 (62.9%)	17 (65.4%)	37 (34.9%)
Age in years, mean ± SD	11.5 ± 2.3	11.6 ± 2.5	12.3 ± 2.5	10.5 ± 2.4
Number of siblings				
0	20 (27.0%)	6 (8.6%)	0 (0%)	10 (9.4%)
1	30 (40.5%)	34 (48.6%)	18 (69.2%)	45 (42.5%)
2	17 (23.0%)	24 (34.3%)	5 (19.2%)	40 (37.7%)
3 or more	7 (9.5%)	4 (5.7%)	3 (11.5%)	7 (6.6%)
Missing data	0 (0%)	2 (2.9%)^#^	0 (0%)^#^	4 (3.8%)
Education				
Primary school	31 (41.9%)	25 (35.7%)	9 (34.6%)	61 (57.5%)
Secondary modern school	18 (24.3%)	19 (27.1%)	9 (34.6%)	19 (17.9%)
Secondary school	22 (29.7%)	12 (17.1%)	7 (26.9%)	23 (21.7%)
Special school	3 (4.1%)	10 (14.3%)	0 (0%)	1 (0.9%)
Missing data	0 (0%)	4 (5.7%)^#^	1 (3.8%)^#^	2 (1.9%)^#^

KINDL-R: revised KINDer Lebensqualitätsfragebogen; HBD: hereditary bleeding disorders (36 patients with hemophilia A and hemophilia B, 22 patients with von Willebrand disease type 2 and type 3, 5 patients with hereditary fibrinogen deficiency, and 9 patients with factor V, VII, and XI deficiency); TIA: transient ischemic attack; # does not sum up to 100% due to rounding errors.

**Table 2:** Differences in self-reported quality of life between patients with hereditary bleeding disorders and their siblings and peers, respectively, measured with the KINDL-questionnaire (overall wellbeing and all subscales); results of hierarchical linear regression models with random intercepts for treatment center.

KINDL-R scores	Patients compared with healthy siblings			Patients compared with healthy peers		
	*β*_*x*_	95%-CI	*p*	*β*_*x*_	95%-CI	*p*
Overall wellbeing	−0.3	(−7.3 to 6.6)	.92	−0.5	(−4.5 to 3.5)	.81
Physical wellbeing	−0.1	(−11.9 to 11.8)	.99	3.3	(−3.8 to 10.4)	.36
Emotional wellbeing	0.9	(−7.3 to 9.0)	.21	0.5	(−4.6 to 5.6)	.84
Self-worth	−2.4	(−16.4 to 11.6)	.74	−6.6	(−14.4 to 1.1)	.09
Family-related wellbeing	0.7	(−9.0 to 10.4)	.88	1.7	(−3.9 to 7.3)	.54
Friend-related wellbeing	0.4	(−11.4 to 12.2)	.38	0.3	(−6.8 to 7.4)	.93
School-related wellbeing	−1.5	(−13.4 to 10.4)	.80	0.2	(−6.7 to 7.1)	.95

KINDL-R: revised KINDer Lebensqualitätsfragebogen; models adjusted for age, gender, number of siblings, and school education.

**Table 3:** Differences in self-reported quality of life between patients with stroke or transient ischemic attack (TIA) and their siblings and peers, respectively, measured with the KINDL-questionnaire (overall wellbeing and all subscales); results of linear regression models.

KINDL-R scores	Patients compared with healthy siblings			Patients compared with healthy peers		
	*β*_*x*_	95%-CI	*p*	*β*_*x*_	95%-CI	*p*
Overall wellbeing	−0.8	(−6.6 to 5.1)	.80	−0.5	(−4.5 to 3.5)	.81
Physical wellbeing	0.8	(−11.0 to 12.6)	.90	−5.1	(−11.7 to 1.5)	.13
Emotional wellbeing	−2.8	(−13.2 to 7.6)	.59	−6.7	(−12.2 to −1.1)	.0185
Self-worth	−1.7	(−3.9 to 0.5)	.13	−8.2	(−13.8 to −2.7)	**.0040**
Family-related wellbeing	−5.7	(−14.4 to 2.9)	.19	−4.4	(−9.6 to 0.7)	.09
Friend-related wellbeing	−5.6	(−17.1 to 5.9)	.33	−13.6	(−19.8 to −7.3)	**<.001**
School-related wellbeing	−0.9	(−11.7 to 9.8)	.86	−7.0	(−13.4 to −0.6)	.0315

KINDL-R: revised KINDer Lebensqualitätsfragebogen; models adjusted for patient group (patients with hereditary bleeding disorders versus patients with stroke/TIA), age, gender, number of siblings, and school education; significant results are shown in bold (*p* < .0071, Bonferroni correction).

**Table 4:** Internal consistency (Cronbach's alpha) of the self-reported KINDL-R questionnaire (overall wellbeing and all subscales) in all patients (HBD and stroke/TIA), their healthy controls, and normative data of 11–17-year-old study participants deriving from the German National Health Interview and Examination Survey for Children and Adolescents, KIGGS.

KINDL-R scores	Patients^#^ (*n* = 144)	Healthy controls^#^ (*n* = 106)	11–17-year-old population based children from the KIGGS-study^$^ (*n* = 7,649)
Overall wellbeing	0.79	0.83	0.82
Physical wellbeing	0.63	0.71	0.59
Emotional wellbeing	0.53	0.51	0.59
Self-esteem	0.63	0.66	0.68
Family-related wellbeing	0.65	0.63	0.72
Friend-related wellbeing	0.60	0.46	0.53
School-related wellbeing	0.51	0.69	0.53

KINDL-R: revised KINDer Lebensqualitätsfragebogen; HBD: hereditary bleeding disorders; TIA: transient ischemic attack. ^#^The number of persons included in the analyses varies slightly between subdimensions, depending on the completeness of the KINDL-R questionnaire; KIGGS: German National Health Interview and Examination Survey for Children and Adolescents. ^$^Data taken from Erhart et al. [12].

## Additional Points

These findings are based on small sample sizes in a cross-sectional study design, all analyses were exploratory, and results should therefore be interpreted with caution. There were no data on nonresponder; thus the representativeness regarding the source population remains unclear. The intercorrelation among test items measured with Cronbach's *α* was good: in all study subgroups overall wellbeing scores showed an acceptable internal consistency (*α* > 0.7). In subdimensions of the KINDL-R score however some subgroups showed poorer results but in the representative KIGGS population sample (only children ≥ 11 years) some subdimensions were also below acceptable internal consistency [12]. The poor internal consistency in healthy controls in one subdimension and the modest reliability in all but one subdimension in both patients and healthy controls may reflect limitations of the questionnaire per se as well as specific limitations of the small sample size in this investigation. When combining all study subgroups Cronbach's *α* was 0.82 and subscales ranged between 0.52 and 0.67 (data not shown). Thus, the use of the generic KINDL-R questionnaire seems applicable in this specific setting. Self-reported measures of health status are regarded as the primary method for assessing HrQoL in children and adolescents once they have reached a certain age and level of cognitive development [24, 25]. Longitudinal data were necessary to elucidate whether adolescents or adults with HBD who showed as children a HrQoL comparable to population norms exhibit over time, relative to the population level, a deterioration of their self-reported Hr-QoL, especially regarding physical wellbeing.
